# The Effects of an Urban Forest Health Intervention Program on Physical Activity, Substance Abuse, Psychosomatic Symptoms, and Life Satisfaction among Adolescents

**DOI:** 10.3390/ijerph15102134

**Published:** 2018-09-28

**Authors:** Riki Tesler, Pnina Plaut, Ronit Endvelt

**Affiliations:** 1 Department of Health System Management, Faculty of Health Science, Ariel University, Ariel 407000, Israel; 2 Faculty of Architecture and Town Planning, Technion-Israel Institute of Technology, Haifa 3200003, Israel; pninatech@gmail.com; 3 School of Public Health, University of Haifa, Haifa 3498838, Israel; rendevelt@univ.haifa.ac.il

**Keywords:** urban forest, at-risk youth, risky behavior, physical activity, intervention, psychosomatic symptoms, life satisfaction

## Abstract

*Background*: At-risk adolescents have been defined as youth who are or might be in physical, mental, or emotional danger. An Urban Forest Health Intervention Program (UFHIP) was formed at a center for at-risk adolescents in Israel, in order to promote physical activity and reduce risky behavior. *Objective*: To evaluate the intervention’s effect on physical activity, smoking, alcohol consumption, psychosomatic symptoms, and life satisfaction. *Methods*: From 2015 to 2016, at-risk youth were nonrandomly selected to participate in the UFHIP. Questionnaires were administered to both intervention and control groups before and after the intervention. Univariate and multivariable analyses evaluated the intervention’s effect. *Results*: The study participants (*n* = 53) showed 0.81 more sessions per week of 60 min of physical activity than did the control group (*n* = 23; *p* = 0.003). Among the intervention group, smoking frequency reduced from a mean of 2.60 (SD = 1.30) to 1.72 (SD = 1.08), whereas that in the control group increased from 3.17 (1.03) to 3.39 (1.03). In both groups, there was a reduction in alcohol consumption, with a greater change among intervention participants: −1.08 (SD = 1.30), compared with −0.09 (SD = 1.79) in the control group. *Conclusions*: Findings indicate that the environmental intervention was efficacious in increasing physical activity and reducing risky behaviors among youth. The effectiveness of this intervention among larger samples is warranted in future prospective studies.

## 1. Introduction

Adolescents deal with a wide variety of tasks and pressures in their lives related to different school, family, and social experiences [1]. Various elements in their environment, such as low socioeconomic status, belonging to a minority group, or exposure to domestic abuse, put adolescents at risk for health-related afflictions [2]. The term “at-risk youth” refers to adolescent populations that are or might be in situations of physical, mental, or emotional risk. In many cases, this population includes teenagers who study in educational settings that are tailored for their specific needs, or adolescents who have dropped out of the formal school system and are exposed to negative social interactions or delinquency, and who develop risky behaviors including smoking cigarettes, abusing drugs, and consuming alcohol [3,4].

Regularly engaging in physical activity increases self-worth and self-efficacy, and has the potential to reduce other risky health behaviors, which is particularly important for at-risk populations [5,6]. It has been established that low levels of and reduction of physical activity can increase the risk of chronic diseases, leading to possible negative behavioral, cognitive, and physiological outcomes [7]. In addition to the health benefits of physical activity, engaging in physical activity in nature settings (e.g., in the forest) has unique benefits [6,8]. One systematic review [8] that compared indoor and outdoor physical activity found that activities in a natural or green environment contribute positively to the quality of relationships with peers and family, as well as to cognitive functioning and the reduction of risky behaviors [9,10,11].

In Israel, specific risky behaviors, such as alcohol consumption and cigarette smoking, are apparent among adolescents. A recent Health Behavior in School-Age Children (HBSC) survey [12] found that among 15-year-olds, 15% of boys and 5% of girls had been drunk on two or more occasions. The rate of 15-year-olds who smoked reached 20% for both boys and girls; as adolescents got older, the rate increased [12]. The legal age for purchasing alcohol and cigarettes in Israel is 18 years old. The initial age of alcohol consumption in Israel is dropping, while the proportion of binge drinking is rising [12,13]. Binge drinking among adolescents is associated with many additional risky behaviors, such as drug abuse and unprotected sexual behavior [14,15,16]. In addition, cigarette smoking has been found to be associated with drinking alcohol; those who smoke more cigarettes are more likely to drink more alcohol [17]. Adolescents who drink alcohol have a lower chance of overcoming smoking addictions [18]. Smoking has also been associated with bullying, being victimized by bullying [19], eating disorders, and low life satisfaction [20,21].

Various mental health problems, such as depression and anxiety, have been found to be associated with physical health problems among children and adolescents. Such physical health problems may include obesity, asthma, and diabetes, as well as harmful habits, such as smoking and alcohol consumption, which might continue into adulthood [18,19,20,21]. Mental health and emotional resilience in adolescence have been found to be associated with reports of good physical health in adulthood [22].

Several personal and environmental factors, such as genetics, behavior, social and community ties, and socioeconomic status [23,24], impact upon and can predict a proper state of health. A strong predictor of good physical and mental health is a positive sense of well-being, which includes positive feelings and future expectations, positive social conception, and high self-esteem [25,26,27]. Feelings of joy, positivity, and high self-esteem have an important role in one’s physical, mental, behavioral, and social functioning [27,28]. There is also a strong relationship between nature activities, a positive sense of well-being, and the functioning of one’s immune, cardiovascular, and neurological systems over time [28,29,30]. A positive sense of well-being, which can be derived from an increase in physical activity among adolescents through outdoor intervention programs [8,9,11,30], has been found to lead to a drop in risk behaviors [26]. In addition to participation in outdoor physical activity, another important factor in adolescents’ physical and mental health is life satisfaction. Compared to those with a low level of life satisfaction, adolescents with a high level of life satisfaction report more positive outcomes in school and in interpersonal and intrapersonal interactions, less depression, and less social stress [31].

### 1.1. Urban Forests and Parks as Potential Spaces for Health Promotion

Forests and parks located in urban surroundings may be an important component in promoting the quality of life and the mental, physical, and social well-being of the community in general, and of adolescents in particular [32,33]. Many studies have shown the considerable potential of establishing forests and parks—open areas devoid of construction that are accessible to the public and contain sufficient territory for conducting active recreational activities [34,35]. Urban green spaces provide optimal environments for partaking in leisure activities and encouraging physical activity while, at the same time, promoting mental well-being [36,37,38]. Moreover, urban forests are a significant and increasingly valuable component of the urban environment. First, these green areas preserve the environment, purify it, and reduce air pollution [32]. Second, the forest contributes to the individual’s sense of social well-being by providing a pleasant and safe environment for forming social interactions and enhancing community cohesiveness [32,33]. Third, the natural environment contributes to psychological health by improving one’s mood and reducing perceived stress [33,39]. Fourth, this environment facilitates the performance of active leisure activities at a frequency and quantity that can attain health goals recommended by public health organizations [35,36,40].

### 1.2. The Urban Forest Health Intervention Program for Encouraging an Active and Healthy Lifestyle

Aside from individual-related factors, there has been an increasing awareness in the last decade of the physical environment of children and adolescents, particularly the existing leisure infrastructure, as a factor influencing their engagement in physical activity [36,37,41].

The idea underlying the Urban Forest Health Intervention Program (UFHIP) sought to meet the need for an adequate physical environment that promotes engagement in active leisure time. Many studies around the world have shown that cooperation between healthcare organizations and forest organizations, with an aim of encouraging physical activity among different groups, is beneficial for all. Such cooperation allows for a high quality of life, as well as for mental, physical, and social well-being through the optimal utilization and design of forests and parks in urban surroundings [30,40].

To date, no comprehensive study has extensively examined overall personal and social factors and their effect on physical activity by means of urban forests. In Israel, there is a dearth of data on physical activity, recreation, and leisure habits in urban forests in general, and for at-risk adolescents in particular. We hypothesized that participation in UFHIP would increase physical activity habits; reduce the frequency of cigarette smoking, alcohol consumption, and psychosomatic symptoms; and increase life satisfaction among at-risk adolescents.

## 2. Materials and Methods

### 2.1. Study Setting and Description of UFHIP

The UFHIP was created as a collaborative effort by several organizations: the Israel Institute of Technology (Technion), the Jewish National Fund, the Ministry of Health, Maccabi Health Services, Clalit Health Services, the University of Haifa, the Israel Anti-Drug Authority, and the municipality of Karmiel. The program took place in Karmiel, a city in northern Israel. There are 46,700 residents in Karmiel, and the city ranks 5 out of 10 in the socioeconomic ranking of Israel’s cities (a rank of 1 being the least affluent and 10 being the most affluent) [42]. UFHIP was conducted at the youth advancement center, a specific organization that provides an educational framework for at-risk children and youth who have dropped out of the formal education system. There is an urban forest (called the Tazahal Forest) in the vicinity of the youth advancement center, about 500 meters from the center, where many activities were held. The adolescents met at the Hila Center five days a week. The intervention program took place three times a week, with activities focusing on physical activity, such as developing physical fitness, bike riding, rope climbing, and hiking. Sessions on nutrition and physical activity were led by experts: a dietician, a psychologist, two physical education teachers, and five counselors. There were additional activities relating to group dynamics and developing personal and group leadership. The students were chosen nonrandomly to participate in the program. Each activity lasted about 60 min, and was led by an instructor who guided the students in the activity at the urban forest and then brought them back to the youth advancement center. After choosing activities for the first session, students were not allowed to switch during the program. Two additional sessions were held for all participants and their families—a joint activity that included nature outings and workshops. During the program, discussions were held with the participants regarding the activity performed and their satisfaction with the program. At the end of the program, a group meeting was held with the entire team of instructors, adolescents, and their families. The program took place between September 2015 and June 2016.

### 2.2. Research Design

**Figure 1:**
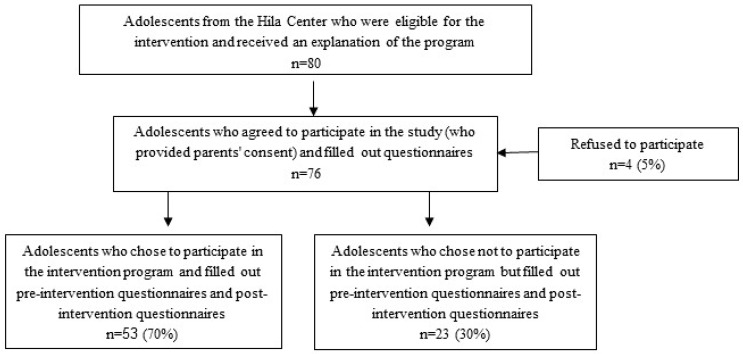
Flow of the study population.

A total of 80 adolescents (out of approximately 100) who studied at the youth advancement center were eligible for the intervention, and received an explanation about the program. Four students refused to participate, leaving 76 who consented (who provided parental consent forms) and filled out questionnaires. Students and parents were able to ask questions about the intervention prior to completing the consent form. A nonrandomized controlled study of the intervention program was performed on these 76 adolescents, of whom 53 were in the intervention group and 23 in the control group (Figure 1). Those in the intervention program filled out questionnaires before and after the intervention program, as well as participating in the intervention program; those in the control group did not participate in the program, but did fill out pre- and post-intervention questionnaires. Pre-intervention questionnaires were administered before the intervention took place in the first week of September 2015, and post-intervention questionnaires took place following the intervention, during the last week of June 2016. Study outcomes were measured before and after the intervention.

### 2.3. Data Collection and Survey Instrument

#### Survey Content

A self-reported student survey, based on the Israeli version of the HBSC questionnaire, was administered to both intervention and control groups before and after the intervention [43]. The original HBSC survey was validated in a previous study [41,44,45]. This survey asked questions about smoking, excessive alcohol consumption, nutritional habits, physical exercise, internet use, life satisfaction, sociodemographic factors (socioeconomic status, ethnicity, gender, age, and school level), and body mass index. Readers are referred to the published study protocol for a detailed overview of the operationalization of the survey items [41,43]. In our version of the questionnaire, we added questions about attitudes, perceptions, and behavioral patterns regarding urban forests. Our questionnaire was validated after adding six additional questions, which produced a Cronbach’s alpha equal to 0.82.

Each questionnaire took each student in the sampled classes 40–50 min to complete. We conducted a pretest study, with 10 students who were randomly selected from the youth advancement center who did not participate in the intervention program, to ensure the validity and reliability of the questionnaire. These students were excluded from the main study. Following the pilot study, we made minor adjustments to the questionnaire, such as deleting unnecessary reply options and simplifying the language so that it was easier for the students to understand. Questionnaires were administered by staff members from the youth advancement center and the research team.

### 2.4. Measures and Instruments

Variables used for the analysis: five dependent variables were tested, and each was measured at the beginning and end of the study. Each questionnaire included 33 questions. The following variables were included:

#### 2.4.1. Physical Activity in the Urban Forest

Physical activity in the urban forest was measured by the participants’ engagement in moderate-to-vigorous physical activity or vigorous physical activity. Moderate-to-vigorous physical activity (MVPA) was defined as any activity that increases heart rate, as defined by the HBSC [41]. Physical activity in the forest could include playing sports, running, walking, biking, or playing with friends. To calculate the amount of time spent on MVPA in the forest, the study and control groups were asked a number of questions, including “Over the past 7 days, how many days were you physically active in the Tazahal forest for a total of at least 60 min a day?” The possible answers included “every day”, “4–6 times a week”, “2–3 times a week”, “once a week”, “once a month”, “less than once a month”, and “never”. Another question was “How many hours a week in your free time do you usually exercise in the Tazahal forest to the extent that you get out of breath or sweat?” The possible answers included “none”, “about half an hour”, “about 1 h”, about 2–3 h”, “about 4–6 h”, and “about 7 h or more”.

Recent studies have supported the validity of self-reported questionnaires regarding physical activity over the past 7 days [44].

#### 2.4.2. Substance Use: Cigarette Smoking

The frequency of cigarette use was measured through the following question: “How often do you smoke tobacco at present?” Participants answered on a four-point scale from 1 (never) to 4 (every day). Self-report smoking measures have been found to have good reliability and validity [46].

#### 2.4.3. Substance Use: Alcohol Consumption

To measure alcohol consumption levels, two questions were asked. The first related to drunkenness: “Have you ever had so much alcohol that you were really drunk?” Answers included “never” (given a rating of 1), “once” (given a rating of 2), “2–3 times” (given a rating of 3), “4–10 times” (given a rating of 4), and “more than 10 times” (given a rating of 5). A second question referred to binge drinking: “In the past 30 days how many times have you drunk five alcoholic drinks or more within a period of a few hours?” Answers included “never” (rating of 1), “not in the past month” (rating of 2), “once” (rating of 3), “twice” (rating of 4), “three times” (rating of 5), and “four times or more” (rating of 6). HBSC items on drunkenness and binge drinking have been well used and found to have good predictive and criterion validity [45,47].

#### 2.4.4. Psychosomatic Symptoms

Psychosomatic symptoms were assessed by the eight-item HBSC psychosomatic symptom checklist [48]. In the checklist, participants were asked how often they had experienced the following symptoms in the past six months: headaches, stomach aches, backaches, feeling low, irritability or bad temper, feeling nervous, difficulties in getting to sleep, and feeling dizzy. Answers included “rarely or never” (1), “about every month” (2), “about every week” (3), “more than once a week” (4), and “about every day” (5). The HBSC psychosomatic symptoms list has been widely used as a proxy for adolescent psychological well-being [48]. An overall psychosomatic symptoms index combining all items was created (Cronbach’s alpha = 0.86).

#### 2.4.5. Life Satisfaction

Life satisfaction was measured by a single item that was ranked on a 0 to 10 score, with 10 representing higher well-being and satisfaction. Participants were asked to rate their life satisfaction using a visual analog scale, the “Cantril ladder”, with 10 steps (10 = best possible life; 1 = worst possible life) and to indicate where on the ladder they would place their present life. A score of 6 or more indicated high life satisfaction [49]. This item has been used in previous work and has shown good reliability and significant associations with other well-being measures [49].

### 2.5. Data Analyses

Differences between the groups in continuous variables were tested using the independent samples *t*-test. Categorical variables were tested with the chi-square test, and ordinal variables were tested with the Mann–Whitney test. Study outcomes were defined as changes from before the intervention to after it. Differences between the studied group outcomes were tested for significance using the independent *t*-test. All analyses were performed with R version 3.4.2. [50].

### 2.6. Ethical Approval

The research protocol received approval from the ethics committees of the Israel Institute of Technology (Technion). All respondents were given explanations before data collection and were advised that participation was voluntary.

## 3. Results

### Survey Findings

There were 76 at-risk adolescents who participated in the UFHIP; 53 were included in the intervention group and 23 were included in the control group. The mean age of the intervention group was 16.9 years (standard deviation (SD) = 0.89 years); the mean age of the control group was 16.7 years (SD = 0.75). In the intervention group, there were 25 girls and 28 boys; in the control group, there were 10 girls and 13 boys. More descriptive statistics of the study population are presented in Table 1.

We hypothesized that the physical activity level of those adolescents who participated in the intervention program would increase. The results supported the hypothesis in both physical activity measurements. In days during the week with at least one hour of physical activity, individuals from the intervention group showed 0.81 more sessions of 60 min of physical activity per week than the control group (*p* = 0.003). In total physical activity hours that took place after school, intervention group participants showed 1.39 more hours than the control group (*p* < 0.001).

The frequency of smoking decreased in the intervention group from a mean of 2.60 (SD = 1.30) to 1.72 (SD = 1.08), following the program. Among the control group, there was an increase from a mean of 3.17 (1.03) before the intervention to 3.39 (SD = 1.03) after the intervention (*p*-value of the differences between the groups after the intervention was 0.005). Alcohol consumption also decreased in the intervention group from a mean of 3.74 (SD = 1.14) to 2.68 (SD = 0.79). In the control group, it remained similar: 2.99 (SD = 1.54) before and 2.95 (SD = 1.38) after the intervention (*p*-value of the differences between the groups was 0.026). Psychosomatic symptoms decreased more for the intervention group than for the control group, with a change of −1.37 (SD = 0.76) versus −0.18 (SD = 0.70), respectively, with a *p*-value of <0.001. Life satisfaction increased more for the intervention group than for the control group, with a change of 1.42 (SD = 1.99) versus −0.29 (SD = 2.69), respectively, with a *p*-value equal to 0.013 (see Table 2).

## 4. Discussion

Few studies have been conducted on the impact of intervention programs that aim to encourage physical activity among youth as a preventive measure, strengthening their mental health and reducing risk factors that increase the probability of addiction or other problematic behavior on a personal and social level [9,10,11,26]. Findings from the present study reinforce the importance of cooperation between organizations devoted to promoting community health and between partnering organizations in the intervention program. The shared resources of the Israel Institute of Technology (Technion), the Jewish National Fund, the Ministry of Health, Maccabi Health Services, Clalit Health Services, the University of Haifa, the Israel Anti-Drug Authority, and the municipality of Karmiel resulted in a more comprehensive and widespread program than any one organization could accomplish on its own. The joint effort possibly contributed to the positive results of the program.

Our study was based on the following hypotheses. The first hypothesis was that the UFHIP would lead to a change in the frequency of physical activity of at-risk youth. This hypothesis was fully confirmed: the frequency of physical activity among the youth participating in the intervention program was greater than that among the control group. All measures relating to frequency of physical activity before and after the program were significant. Confirmation of the first hypothesis is consistent with findings of other studies and supports the need to promote physical activity habits among children and youth in Israel [42,43]. Physical activity can take place in a variety of environments, including parks that are usually accessible to the public. It is well known and has been established that parks provide good opportunities for promoting physical activity [7,9,10,11,36]. Findings of a recently published review on the association between parks and physical activity indicated a positive relationship between visiting parks and partaking in physical activity [36,37]. One of the studies surveyed found that individuals who frequented parks on a monthly basis had a 4-times-greater probability of engaging in physical activity at least five times per week for longer than half an hour, thereby attaining the recommended level of physical activity per day [40].

The second and third hypotheses suggested that participation in the UFHIP would lead to a change in risk behaviors, including cigarette smoking and excessive alcohol consumption. These hypotheses were fully confirmed in all measures. In the intervention group, there was a significant decrease in the frequency of smoking following the intervention. By contrast, in the control group, there was no significant difference in frequency of smoking before or after the intervention. Regarding excessive alcohol consumption, in the intervention group, there was a significant decrease in the frequency of excessive alcohol consumption before and after the intervention. On the other hand, in the control group, there was no significant difference in the frequency of alcohol consumption in the before and after measures. These findings confirm and strengthen previous studies, which have found that physical activity in forests and parks is a strong predictive factor for reducing and preventing risk behavior among adolescents [9,30,32].

The fourth hypothesis asserted that participation in the UFHIP program would lead to a change in reported psychosomatic symptoms. The findings fully confirmed this hypothesis. In the intervention group, there was a significant difference between the reported level of psychosomatic symptoms measured before and after the program; there were improved reports of psychosomatic symptoms following the intervention. Alternatively, in the control group, there was no significant difference in the reported level of psychosomatic symptoms before and after the intervention period. Various studies have shown both a direct and indirect association between parks and mental health mediated by physical activity, with parks seen as an opportunity for physical activity that can subsequently lead to improved mental health [29,34,35,36].

Our fifth hypothesis stated that participation in the program would lead to a change in the measure of life satisfaction of at-risk youth. In the intervention group, there was a significant difference in the life satisfaction measures before and after the intervention. The average scores indicated improved life satisfaction scores following the intervention compared with scores prior to the program in the intervention group. In the control group, there was no significant difference in the sense of life satisfaction between the before and after measures. Previous studies have investigated the use of parks for leisure and focused on the positive influence of parks on improving mood, relieving stress, and enhancing the sense of life satisfaction and well-being [38,39].

This study had several strengths and limitations. A major strength was that UFHIP was examined in the light of the beneficial interaction between the urban forest and awareness of the influence of physical activity in nature settings. Additionally, as this area of research is relatively new, our study is one of the first to investigate the connection between physical activity in nature settings and reductions in risky behavior among at-risk youth. It is also very likely that since the activities were completed in group sessions, as opposed to individually, there was a high success rate of the intervention program. Finally, another factor that may have led to the program’s success is due to the fact that activities were held in an open, nature setting, as opposed to a school or gym.

This study included some limitations. First, as the adolescents who attended the youth advancement center have dropped out of the formal school system, many face different challenges and have emotional difficulties as compared to their peers in the formal school system and, therefore, may have felt threatened by the process of completing the questionnaires. Some adolescents also had difficulty reading and comprehending the questions and needed help. Additionally, data were self-reported and subject to error, including respondents’ overreporting of socially desirable responses (social desirability response bias). Finally, it would have strengthened our study to include an objective measure (such as a pedometer or heart rate monitor watch) to assess physical activity levels during the study period.

## 5. Conclusions

The results of this study indicate that at-risk youth are more likely to use urban forests for physical activity than the control group. We also found that the frequency of smoking and alcohol consumption among at-risk youth declined among program participants following the intervention program. Urban forests are important spaces for the overall development and physical activity of children and adolescents. Research on children’s use of urban forests is lacking, particularly studies based on direct observation as opposed to parental reports. Since urban forests are associated with increased physical activity and decreased risky behavior, and are generally widely available in urban areas, we recommend future research to focus on the social and environmental characteristics that stimulate higher levels of urban forest physical activity. We believe that not only should at-risk youth benefit from the use of urban areas for physical activity, but that this concept should also be implemented in broader settings, such as in educational and health systems.

## Figures and Tables

**Table 1:** Demographic background of study and control groups (*n* = 76).

Variable	Parameter/Value	Intervention Group *n* = 53	Control Group *n* = 23	Total *n* = 76	*p*-Value
Age	Mean (SD)	16.9 (0.89%)	16.7 (0.75%)	16.9 (0.85%)	0.367 ^a^
Gender	Male	28 (53%)	13 (57%)	41 (54%)	0.767 ^b^
Country of origin	Israel	44 (83%)	18 (78%)	62 (82%)	0.623
Level of religiosity	Secular	38 (72%)	13 (57%)	51 (67 %)	0.212 ^c^
	Traditional	13 (25%)	9 (39%)	22 (29%)	
	Religious	2 (4%)	1 (4%)	3 (4%)	
Socioeconomic status	Low	7 (13%)	5 (22%)	12 (16%)	0.845
	Average	36 (68%)	11 (48%)	47 (62%)	
	Good	9 (17%)	7 (30%)	16 (21%)	
	Very good	1 (2%)	0	1 (1%)	
Mother’s education	Elementary	0	0	1 (1%)	0.298
	Middle school	2 (4%)	1 (5%)	4 (6%)	
	High school	24 (42%)	13 (52%)	31 (42%)	
	Professional *	14 (26%)	6 (26%)	20 (26%)	
	Academic	15 (28%)	4 (17%)	19 (25%)	
Father’s education	Elementary	1 (2%)	0	1 (1%)	0.971
	Middle school	4 (7%)	1 (5%)	5 (7%)	
	High school	19 (36%)	11 (50%)	30 (40%)	
	Professional *	18 (34%)	4 (18%)	22 (29%)	
	Academic	11 (21%)	6 (27%)	17 (23%)	

Abbreviations: SD, standard deviation; ^a^ Continuous variables were tested with independent sample *t*-testing; ^b^ Categorical variables were tested with the chi-square test; ^c^ Ordinal variables were tested with the Mann–Whitney *U* test; * Bachelor's degree or higher.

**Table 2:** Differences between groups in the changes before and after intervention.

Variables	Before Intervention		After Intervention		Change		*p*-Value
	Study Mean (SD)	Control Mean (SD)	Study Mean (SD)	Control Mean (SD)	Study Mean (SD)	Control Mean (SD)	
60 min PA sessions in a week	2.28 (0.80)	1.74 (0.81)	3.34 (1.21)	2.00 (1.04)	1.07 (1.44)	0.26 (0.81)	0.003
Total after school PA hours	2.71 (1.11)	2.26 (1.25)	4.21 (1.26)	2.39 (1.34)	1.52 (1.75)	0.13 (1.06)	<0.001
Frequency of smoking	2.60 (1.30)	3.17 (1.03)	1.72 (1.08)	3.39 (1.03)	−0.92 (1.57)	0.22 (1.51)	0.005
Alcohol consumption	3.74 (1.14)	2.99 (1.54)	2.68 (0.79)	2.95 (1.38)	−1.08 (1.30)	−0.09(1.79)	0.026
Psychosomatic symptoms	2.91 (0.78)	2.05 (0.45)	1.54 (0.43)	1.94 (0.60)	−1.37 (0.76)	−0.18(0.70)	<0.001
Life satisfaction	6.21 (1.75)	6.82 (2.28)	7.62 (1.01)	6.45 (2.46)	1.42 (1.99)	−0.29(2.69)	0.013

Abbreviations: SD, standard deviation.
